# Fasting serum potassium and long-term mortality in healthy men

**DOI:** 10.1186/s12889-021-10738-4

**Published:** 2021-04-13

**Authors:** Ragnhild S. Falk, Trude Eid Robsahm, Jan Erik Paulsen, Tanja Stocks, Isabel Drake, Trond Heir

**Affiliations:** 1 Oslo Centre for Biostatistics and Epidemiology, Oslo University Hospital, Pb 4950 Nydalen, N-0424 Oslo, Norway; 2 Department of Research, Cancer registry of Norway, Institute of Population Based Cancer Research, Oslo, Norway; 3 Department of Food Safety and Infection Biology, Norwegian University of Life Sciences, Oslo, Norway; 4 Department of Clinical Sciences Lund, Lund University, Lund, Sweden; 5 Department of Clinical Sciences in Malmö, Lund University, Malmö, Sweden; 6 Institute of Clinical Medicine, University of Oslo, Oslo, Norway

**Keywords:** Cancer, Cell proliferation, Mortality, Potassium levels, Prospective cohort study

## Abstract

**Background:**

Serum potassium levels have been positively associated with cardiovascular mortality, but little is known about the association with cancer mortality and death due to other causes. We examined whether serum levels of potassium were associated with long-term mortality in a healthy cohort.

**Methods:**

*Oslo Ischemia Study* invited 2341 initially healthy men aged 40–59 years with no use of medication to a comprehensive health survey in 1972. Fasting serum level of potassium (mmol/L) was ascertained at baseline for 1989 men. We have complete follow-up for death throughout 2017. Cox proportional hazard models were used to calculate hazard ratios (HRs) with 95% confidence intervals (CIs) and adjusted for multiple confounders.

**Results:**

After a median follow-up of 30 years (interquartile range 21.2–38.7), 1736 deaths were observed, of which 494 were cancer deaths, 688 cardiovascular deaths, and 536 deaths related to other causes. Restricted cubic spline analysis showed that potassium level was linearly and positively associated with long-term cancer mortality; HR per mmol/L 1.8, 95% CI 1.4–2.4. Compared with low levels of potassium (≤ 4.0 mmol/L), men with high levels (≥4.6 mmol/L) showed a significantly 78% higher risk of cancer death. A positive linear association was found for all-cause mortality (HR per mmol/L 1.6, 95% CI 1.4–1.8), and for cardiovascular (HR per mmol/L 1.4, 95% CI 1.1–1.7) and other cause mortality (HR per mmol/L 1.7, 95% CI 1.3–2.2).

**Conclusions:**

These findings suggest that serum potassium level appears to predict long-term mortality in healthy middle-aged men, and it might imply future surveillance strategies for individuals with high serum potassium levels.

**Supplementary Information:**

The online version contains supplementary material available at 10.1186/s12889-021-10738-4.

## Background

Potassium is an electrolyte that plays an essential role in several intracellular regulating mechanisms. In normal physiology, serum potassium levels are maintained within the range of 3.5 and 5 mmol/L [1]. Potassium levels outside this range have been associated to various diseases such as heart failure, chronic kidney disease and diabetes mellitus [2]. The importance of serum potassium in the cancer process is not well studied in the general population. We have recently reported results showing a linear association between serum potassium and cancer risk [3]. Moreover, higher pre-diagnostic serum potassium has been associated with poorer prognosis in patients with prostate cancer [4]. Knowledge about potassium and cancer mortality is scarce and grounded in studies with few events and limited follow-up. Also, the findings showed both significant and non-significant positive associations [5–7]. Furthermore, the long-term relationship has not yet been investigated in a healthy population.

The importance of potassium in cardiovascular mortality or all-cause mortality has been examined more extensively. Studies have shown a positive association between potassium levels and cardiovascular mortality in the general population [8], and conflicting results in relation to all-cause mortality [5–7, 9–11].

In the current study we aimed to examine the association between fasting serum levels of potassium in initially healthy middle-aged men and long-term cancer mortality. In addition, we aimed to assess the association between potassium and cardiovascular death, other causes of death, and all-cause death.

## Methods

### Data sources

*The Oslo Ischemia Study* is a comprehensive health survey established in 1972, aimed to examine risk factors for coronary heart disease and other cardiovascular diseases in a healthy middle-aged male population. A total of 2341 male employees in five companies with occupational health service in Oslo, Norway, aged 40–59 years, were invited to participate. Inclusion in the study required the absence of any medical diagnosis and no medication use for the past 14 days. Among those invited, 2014 (86%) men agreed to participate and completed the study protocol. At inclusion, all participants underwent a comprehensive review of the medical history, clinical and physical examinations (including measurements of body height and weight), a panel of blood tests, and a maximal exercise tolerance bicycle test. Information on lifestyle variables (e.g. smoking habits) was collected with a questionnaire. Detailed selection and implementation procedures have been reported previously [3, 12–14].

Information about vital status and cause of death were obtained by linkage to the Cause of Death Registry, using the 11-digit personal identification number assigned all Norwegian citizens. The Cause of Death Registry covers all deaths in Norway (i.e reporting is mandatory by law) including deaths of Norwegians who died abroad. A physician determined the cause of death according to the current revision (7th, 8th, 9th or 10th) of the World Health Organization’s *International Classification of Diseases* (ICD). The underlying cause of death was used to identify deaths related to cancer (*Neoplasms; ICD-10 C00-D48*), cardiovascular disease (*Diseases of the circulatory system; ICD-10 I00-I99*) and other causes (details in supplementary Table 1).

At inclusion in the 1970s, all participants provided informed consent according to the Declaration of Helsinki. After increased awareness of privacy during the 1990s, the Data Protection Authority retrospectively reviewed the inclusion process for the Oslo Ischemia Study and judged participation to be in accordance with provisions on written informed consent. This study was approved by the Regional Committees for Medical and Health Research Ethics, Norway.

### Variables

Blood samples for measurement of electrolytes were collected after at least 12 h fasting and 8 h abstaining from smoking. Repeated analysis of the blood samples has shown high accuracy [12]. In the present study, the variable of interest was serum potassium concentration. The continuous level of potassium was given per 1 unit (mmol/L) and per 1 standard deviation (SD) increase. Potassium levels below or above the normal range were categorized as hypokalemia (≤3.5 mmol/L) or hyperkalemia (> 5.0 mmol/L), respectively. The normal range of potassium was divided into three equally spaced categories (normo-low [3.6–4.0], normo-intermediate [4.1–4.5], and normo-high [4.6–5.0 mmol/L]). Due to the small number of observations in the hypo- and hyperkalemia range, potassium levels were also categorized as (low [≤4.0], middle [4.1–4.5], and high [≥4.6 mmol/L]).

The erythrocyte sedimentation rate (mm/hour) was determined by the Westergren method. In addition, fasting blood glucose (mmol/L), serum creatinine (μmol/L), triglyceride (mmol/L), total cholesterol (mmol/L) and sodium (mmol/L) were measured. Body mass index (BMI) was calculated based on the individual’s measured body height and weight at baseline (kg/m^2^). Blood pressure (mm Hg) was measured in the supine position, with a manual mercury sphygmomanometer. Physical fitness was measured as the total work (i.e. sum of the work performed in the bicycle test) divided by body weight (kJ/kg). Information on smoking habits was self-reported from a questionnaire asking about present and past smoking habits, categorized as “never”, “former”, or “present” smokers. We had complete variable information in all subjects except for fasting blood glucose and sodium, which was missing for 5 and 88 men, respectively. Of the 2014 participants, we excluded 15 men due to cancer diagnosis prior to baseline and 10 men for whom values for potassium were not obtained. Thus, 1989 men remained in the study sample for statistical analyses.

The primary outcome was cancer mortality. Further, cardiovascular death, death related to other causes and all-cause mortality were studied as secondary outcomes. The cohort of men was followed for death throughout 2017.

### Statistical analyses

The absolute risk of death, cause-specific and all-causes, was presented as age-adjusted mortality rates per 1000 person-years. Graphically, the cumulative mortality by cause of death was presented for potassium in three categories, during 45 years of follow-up.

The relative risk of death was explored by using Cox proportional hazard models with age as the time scale. Results were presented as hazard ratios (HR) with 95% confidence intervals (CI). Adjustment was performed for BMI, systolic blood pressure, physical fitness, fasting blood glucose, sodium, triglycerides, cholesterol, creatinine, and sedimentation rate. Variables included in the multivariable model were chosen a priori based on potential confounding effects on the association between serum potassium and mortality, and what have been adjusted for in previous studies [5–11, 15, 16]. The proportionality assumption was checked using Schoenfeld residuals. The models were stratified by smoking to allow for the difference in baseline hazards between never, former and present smokers. Likelihood ratio tests for interactions between potassium and the covariates yielded no significant findings. We tested for trend by modeling the variables as continuous. To illustrate the trends in HR over levels of potassium, restricted cubic spline function was performed with four knots chosen according to Harrell’s recommended percentiles [17].

To exclude the possibility that undetected morbidity had caused increased potassium (i.e. reverse causality) we performed restricted analysis, excluding the first 10 years of observation (including 122 deaths), leaving 1867 men in the analysis. Further, to explore the importance of smoking as effect modifier, we stratified the analyses of trends in HR by smoking status.

To overcome the difficulties in interpretation of HRs [18], we also calculated restricted mean survival times (RMST) as a supplement. The RMST at time *t* can be interpreted as the *t*-year life time expectancy [19] and were thus calculated for all-cause deaths. The RMST model was adjusted for the same variables as in the Cox model. The difference in RMST for middle and high levels was compared to low potassium levels for every 10 year (*t* = 10, 20, 30, 40 years) of follow-up.

All statistical tests were 2-sided, with a significance level of .05. All statistical analyses were performed using Stata, version 15 (StataCorp LLC).

## Results

Baseline characteristics of the study cohort according to potassium level are presented in Table 1. The mean age at inclusion was 49.8 years (SD 5.5) and the mean potassium level was 4.3 mmol/L (SD .35). Nineteen men (1%) had hypokalemia (≤3.5 mmol/L), 50 men (3%) had hyperkalemia (> 5.0 mmol/L), and the remaining 1920 men had normokalemia (normo-low 17%; normo-intermediate 58%; normo-high 22%) (Table 2).

**Table 1:** Baseline characteristics of the 1989 men in the study cohort by potassium level

Characteristic	Potassium level		
	Low (≤4.0 mmol/L)	Middle (4.1–4.5 mmol/L)	High (≥4.6 mmol/L)
Age (years)	48.6 (5.5)	49.8 (5.5)	50.7 (5.2)
Height (cm)	177 (6.4)	177 (6.2)	177 (6.1)
Weight (kg)	77.0 (10)	77.3 (9.7)	75.4 (10)
Body mass index (kg/m^2^)	24.6 (2.8)	24.7 (2.7)	24.2 (2.7)
Physical fitness (kJ/kg)	1.53 (.63)	1.43 (.56)	1.37 (.52)
Systolic blood pressure (mm Hg)	133 (19)	129 (17)	130 (17)
Sodium (mmol/L)^a^	141 (3.5)	141 (3.4)	142 (3.7)
Fasting blood glucose (mmol/L) ^a^	4.5 (.60)	4.5 (.54)	4.4 (.54)
Triglycerides (mmol/L)	1.28 (.66)	1.37 (.76)	1.25 (.63)
Cholesterol (mmol/L)	6.5 (1.2)	6.7 (1.2)	6.6 (1.3)
Creatinine (μmol/L)	81.3 (14)	82.2 (14)	82.2 (14)
Sedimentation rate (mm/hour)	6.3 (5.2)	7.3 (6.6)	8.2 (7.4)
Smoking [n (%)], Never	116 (33)	278 (24)	106 (22)
Former	125 (36)	374 (32)	120 (25)
Present	108 (31)	503 (44)	259 (53)

Number are presented as mean and standard deviation if not otherwise specified

^a^Missing information for fasting blood glucose (*n* = 5) and sodium (*n* = 88)

**Table 2:** Number of deaths (cause-specific and all-cause), person-years and age-adjusted mortality rates among initially healthy men during 45 years of follow-up, by fasting level of potassium (mmol/L) at baseline

	Men	Pyrs of follow-up	Cancer death		Cardiovascular death		Other causes of death ^**a**^		All-cause^**b**^	
			Deaths	Rate per 1000 pyrs^**c**^	Deaths	Rate per 1000 pyrs^**c**^	Deaths	Rate per 1000 pyrs^**c**^	Deaths	Rate per 1000 pyrs^**c**^
	No.	No.	No.	95% CI	No.	95% CI	No.	95% CI	No.	95% CI
All	1989	58,329	494	8.5 (7.7–9.3)	688	11.8 (10.9–12.7)	536	9.2 (8.4–10.0)	1736	29.8 (28.4–31.2)
Hypokalemia (≤3.5)	19	593	2	3.3 (.5–23.5)	10	19.8 (5.6–82.5)	4	7.1 (1.8–28.5)	16	30.3 (11.5–96.6)
Normo-low (3.6–4.0)	330	10,751	61	6.2 (3.8–10.3)	106	11.2 (7.7–16.2)	87	8.7 (5.7–13.2)	255	26.1 (20.5–33.4)
Normo-intermediate (4.1–4.5)	1155	33,988	299	8.8 (7.0–11.0)	405	11.9 (9.8–14.5)	303	8.9 (7.1–11.2)	1020	30.0 (26.6–34.0)
Normo-high (4.6–5.0)	435	11,852	144	9.4 (6.5–13.5)	152	11.9 (8.6–16.5)	128	10.4 (7.3–14.8)	397	31.9 (26.2–38.9)
Hyperkalemia (> 5)	50	1146	18	11.8 (5.3–26.9)	15	13.7 (4.7–42.1)	14	9.9 (4.1–24.4)	48	36.0 (20.1–70.8)

*CI* confidence interval, *pyrs* person-years

^a^Death related to other causes than cancer and cardiovascular disease (see supplementary Table 1 for further details)

^b^The all-cause analysis includes 18 men without a specified cause of death

^c^Age-adjusted according to the age-distribution (5-year groups) in the total cohort at baseline

The level of potassium was positively associated with age, sedimentation rate, sodium, and the presence of smoking and was negatively associated with fitness (Table 1). After a median follow-up of 30.0 years (interquartile range 21.2–38.7) there were 1736 deaths, including 494 cancer deaths, 688 cardiovascular deaths, 536 deaths related to other causes, and 18 with missing information about cause of death.

**Fig. 1:**
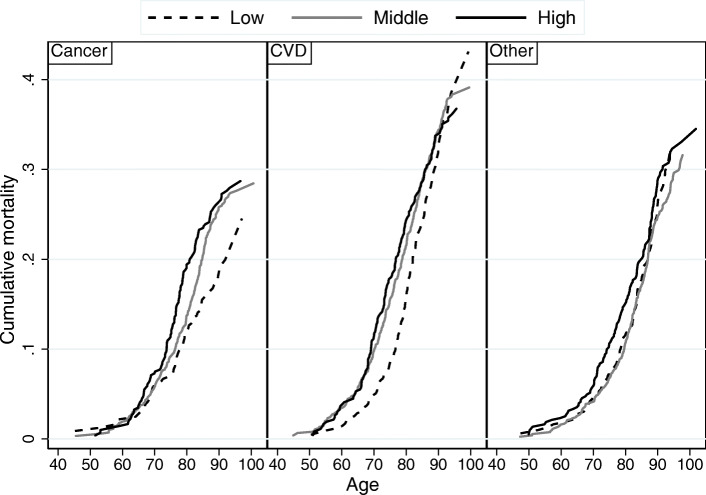
Cumulative cause-specific mortality by baseline level of serum potassium. The cumulative mortality was significantly different among the three categories of potassium (low [≤4.0], middle [4.1–4.5], and high [≥4.6] mmol/L) for cancer deaths (*P* value .010 middle vs low; *P* value .003 high vs low), but not for cardiovascular death (CVD) and other causes of deaths

**Fig. 2:**
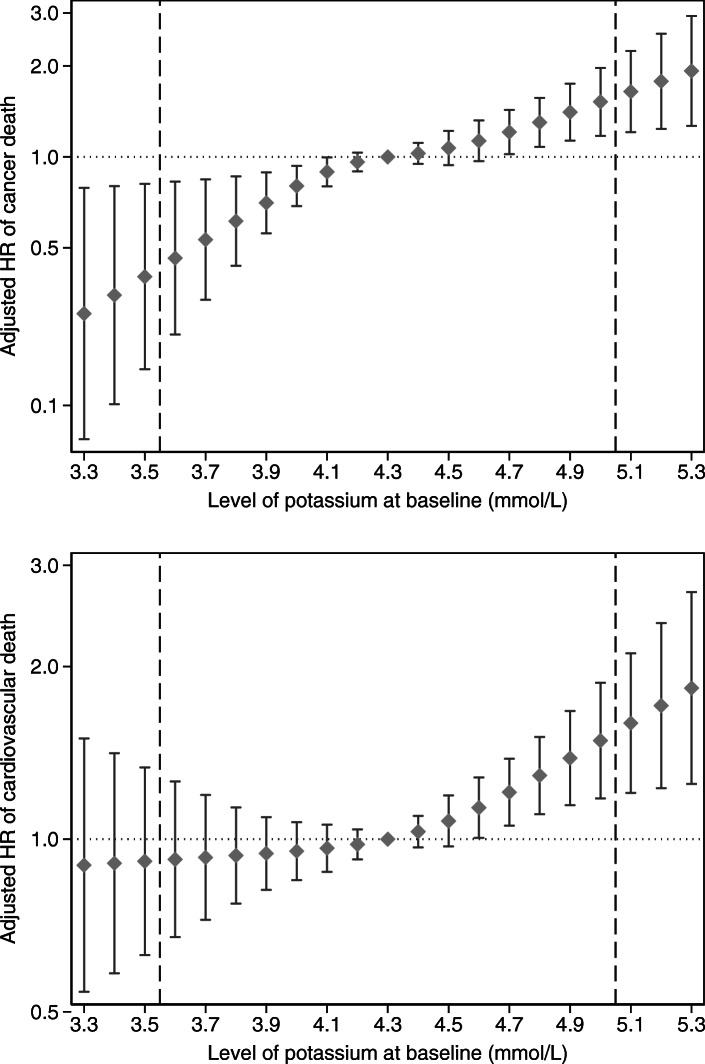
Multivariable adjusted hazard ratio (HR) for **a** (upper panel) cancer death and **b** (lower panel) cardiovascular death by levels of potassium at baseline. Bars indicate 95% confidence intervals. Restricted cubic spline function of potassium with four knots chosen according to Harrell’s recommended percentiles. The reference group was set to the middle value, K = 4.3 mmol/L. Estimates for values ≤3.2 and ≥ 5.4 were not presented due to low numbers. Dashed lines indicate the cut-off values for hypokalemia (≤3.5) and hyperkalemia (> 5.0). Hazard ratios were adjusted according to the multivariable models as presented in Table 3

The age-adjusted mortality rates for death related to cancer increased linearly by increasing potassium level at baseline (Table 2). The differences in cumulative cancer mortality between the three categories of potassium were seen from age 75 years (Fig. 1). When studying hazard ratio of cancer mortality by Cox regression analysis, a linear positive association with potassium level was observed (Fig. 2a). The adjusted HR was 1.22 (95%CI, 1.11–1.35) per SD increase in potassium level, and 1.78 (95%CI, 1.35–2.35) for each mmol/L increase. Compared with low potassium levels (≤4.0, reference group), adjusted HR was 1.53 (95%CI, 1.16–2.02) for middle levels of potassium and 1.78 (95%CI, 1.30–2.42) for higher levels (*p*-value for linear trend < 0.001, Table 3). Restricted analysis, excluding the first 10 years of the study period, did not affect the findings of the regression analyses (Table 3). Stratified by smoking status at baseline, we observed that the association was significant for current smokers, but not for never and former smokers (supplementary Fig. 1).

**Table 3:** Hazard ration (HR) with 95% confidence intervals (95% CI) for cause-specific and all-cause mortality among initially healthy men during 45 years of follow-up, by fasting level of potassium at baseline

Potassium level	Cancer death		Cardiovascular death		Other causes of death^d^		All-cause^e^	
	Age adjusted^**a**^ HR (95% CI)	Multivariable adjusted^**b**^ HR (95% CI)	Age adjusted^**a**^ HR (95% CI)	Multivariable adjusted^**b**^ HR (95% CI)	Age adjusted^**a**^ HR (95% CI)	Multivariable adjusted^**b**^ HR (95% CI)	Age adjusted^**a**^ HR (95% CI)	Multivariable adjusted^**b**^ HR (95% CI)
Per mmol/L	1.90 (1.47–2.46)	1.78 (1.35–2.35)	1.41 (1.13–1.76)	1.37 (1.08–1.74)	1.67 (1.30–2.15)	1.65 (1.26–2.17)	1.62 (1.41–1.87)	1.57 (1.35–1.82)
Per SD^c^	1.25 (1.14–1.37)	1.22 (1.11–1.35)	1.13 (1.04–1.22)	1.11 (1.03–1.21)	1.20 (1.10–1.31)	1.19 (1.08–1.31)	1.18 (1.13–1.24)	1.17 (1.11–1.23)
*Excluding first 10 years of follow-up*, per SD^c^	1.25 (1.14–1.37)	1.23 (1.11–1.36)	1.12 (1.03–1.21)	1.11 (1.02–1.21)	1.20 (1.10–1.31)	1.20 (1.09–1.32)	1.18 (1.12–1.24)	1.17 (1.11–1.24)
Low (≤4.0 mmol/L)	1.00 (ref.)	1.00 (ref.)	1.00 (ref.)	1.00 (ref.)	1.00 (ref.)	1.00 (ref.)	1.00 (ref.)	1.00 (ref.)
Middle (4.1–4.5 mmol/L)	1.64 (1.25–2.16)	1.53 (1.16–2.02)	1.20 (.98–1.48)	1.16 (.94–1.44)	1.17 (.93–1.48)	1.16 (.91–1.48)	1.31 (1.14–1.50)	1.25 (1.09–1.44)
High (≥4.6 mmol/L)	2.00 (1.48–2.70)	1.78 (1.30–2.42)	1.36 (1.08–1.73)	1.24 (.96–1.60)	1.52 (1.17–1.98)	1.41 (1.07–1.86)	1.57 (1.35–1.83)	1.43 (1.22–1.68)
*P* value for linear trend		*<.001*		*.10*		*.01*		*<.0001*

^a^Cox proportional hazards model assuming different baseline hazards for never, former and present smokers with age as the time scale

^b^Additional adjustments for body mass index, systolic blood pressure, fitness, fasting blood glucose, sodium, triglycerides, cholesterol, creatinine and sedimentation rate, all measured at baseline

^c^The standard deviations (SD) for potassium was .35 mmol/L

^d^Death related to other causes than cancer and cardiovascular disease (see supplementary Table 1 for further details)

^e^The all-cause analysis includes 18 men without a specified cause of death

Regarding cardiovascular mortality, the adjusted HR showed no association with low or intermediate levels of potassium, while we observed a positive and linear association for higher potassium levels (Fig. 2b). The association was barely significant when considered as per SD increase (adjusted HR 1.11, 95%CI, 1.03–1.21), and there was no association with potassium as a categorical variable (*P* value for linear trend = .10) (Table 3).

Potassium was positively associated with deaths related to other causes than cancer and cardiovascular disease, although there were slightly lower effect estimates than for cancer mortality (Table 3, supplementary Fig. 2). One third of the deaths was related to diseases of the respiratory system, otherwise a broad specter of causes was observed (supplementary Table 1).

All-cause mortality rates were lowest in men with normo-low potassium levels (3.6–4.0 mmol/L) (Table 2). A trend of increasing mortality was observed for higher levels of potassium (Table 3). The RMST analysis showed consistent results; compared with a low potassium level, men with high potassium levels had a life expectancy for the next 30 and 40 years that was reduced by 1.3 (95%CI, − 2.2 to − 0.4) and 2.5 (95%CI, − 3.7 to − 1.2) years, respectively (supplementary Table 2 and supplementary Fig. 3). No significant differences were observed between low and middle levels of potassium.

## Discussion

In initially healthy middle-aged men we found a positive and linear association between fasting baseline levels of potassium and long-term cancer mortality. The association was evident after adjusting for a series of baseline characteristics. For cardiovascular mortality, we observed a positive and linear association in the higher end of potassium levels. For death related to other causes, we found a positive linear relationship, similarly to that found for cancer mortality.

### Cancer mortality

Our results are consistent with the main findings of three previous studies based on data from the general population [5–7]. Hughes-Austin et al. reported higher cancer mortality (HR 1.2, 95%CI, 1.0–1.5) in individuals with 4.5–4.9 mmol/L compared to those with 4–4.4 mmol/L, and a similar, but non-significant, increase was also observed for levels ≥5.0 [5]. Fang et al. reported a non-significant increasing trend in cancer mortality by serum potassium level [6]. The study by Wannamethee et al. found an increased cancer mortality (HR 1.8, 95% CI, 1.0–3.2) with higher levels of potassium (≥5.2 mmol/L) compared to lower levels (< 5.2 mmol/L), restricted to current smokers [7]. Our results add to previous studies by examining the continuous association in individuals free of any medication, and by extending follow-up to a median of 30 years.

Based on this cohort, we have previously shown a positive and linear relationship between serum potassium and cancer incidence through 30 years of follow-up [3]. The fact that we in the current study observe similar findings for cancer mortality, strengthens the conclusions of both studies. Most likely, the differences in cancer mortality are the result of different risk of being diagnosed with cancer.

We previously proposed three hypotheses that could explain the relationship between potassium and cancer [3]. Firstly, higher level of serum potassium may promote cancer development and growth through, for example immune mechanisms such as suppressing the T-cell activity [20]. Secondly, there may be some inherent individual factors that contribute to the regulation of serum potassium that also facilitate cancer processes. For example, some genetic variations in ion channels and pumps that are involved in potassium homeostasis appear to be associated with genes that have a role in cell proliferations and differentiation. Thirdly, a reverse causation may be observed if necrotic cancer cells - in an undiscovered cancer at baseline - released potassium extracellularly. However, a persistent increase in cancer mortality after excluding the first 10-years of observation reduces the likelihood of the last hypothesis.

### Cardiovascular mortality

Hoppe et al. have performed a systematic review and meta-analysis of observational studies assessing the association of abnormal serum potassium levels and cardiovascular mortality in the older general population. They reported, combined results of six studies, that high potassium levels were associated with a 1.4-fold increased cardiovascular mortality, while no association was detected for low potassium levels [8]. Our results, as shown in Fig. 2b, were in line with their findings, although the results seem to be less clear. Relevant to this comparison maybe that our men were completely medication free at baseline, and that they found the strongest association with higher potassium among users of diuretics [5, 6]. Possible reasons for the increased cardiovascular mortality could result at least partly from fatal ventricular arrhythmias in the course of hyperkalemia [8].

### Mortality from other causes

The positive linear association between potassium and death due to other causes is in accordance with the two previous publications, that observed a linear association [7], and an increased risk within the normal range (HR 1.5, 95%CI, 1.1–1.9 for 4.5–4.9 vs. 4.0–4.4 mmol/L) [5].

### All-cause mortality

Regarding all-cause mortality, conflicting results have been reported, with a linear association [7, 9, 15], a U-shaped association [10, 16], association only for high potassium levels [5, 6], and no association [11] has been observed. The study most similar to ours is a large population-based Swedish cohort study, including healthy individuals in early mid-life, with a median follow-up of 29 years and with fasting measures of serum potassium [9]. They reported a linear association between potassium, per mmol/L increase, and all-cause mortality, HR 1.2, 95%CI, 1.1–1.3. Further, an interaction with serum sodium was found. We observed a slightly stronger linear association, HR 1.6, 95%CI, 1.4–1.8, when potassium was considered continuous, which was most pronounced for high potassium levels. We did not reveal any interaction with sodium. In addition to what was found in the Swedish study, we present RMST for all-cause mortality. The RMST quantified that the average life-years lost was 1.3 and 2.5 years in men with high potassium levels compared with low levels during 30 and 40 years, respectively.

### Clinical implications

We demonstrate that abnormal values of potassium are not common in a healthy population. Although our study suggests that normo-high levels and hyperkalemia may lead to increased mortality. Our data are observational and cannot demonstrate causality, but the results highlights need for further studies on the subject of mechanisms linking serum potassium to mortality.

### Strengths and limitations

There are certain strengths and limitations to our study. The *Oslo Ischemia cohort* is a comprehensively measured and apparently healthy population-based cohort without use of any medication during the last 14 days that has been followed for death through complete national registries for over 40 years, with no loss to follow-up. The cohort has shown similar cancer incidence to men of the same age, time-period and counties of residence [13]. Measurements of potassium were performed fasting, thus minor bias due to potassium intake was expected. However, as a limitation, the potassium level was measured at baseline only, which may both involve measurement error and imprecise estimates of long-term levels, thus leading to diluted association estimates in our study [21]. Outcomes could occur up to 45 years later. Thus, we report not on the immediate impact of potassium levels but the long-term impact by some inherent individual factors, which could be mediated by other events, such as changes in lifestyle variables. However, any bias by this would have been toward null. Residual confounding is a concern in all observational studies, and cannot be entirely ruled out. E.g. information about alcohol consumption would have been preferable. Smoking was self-reported and may suffer from errors in accuracy. We have adjusted for many potential confounders, which did not alter the associations. The strength of the association (risk ratio) of an unmeasured confounder with both the exposure and the outcome, as measured by the E-value, would have to be 1.6 to explain away the observed association between potassium (per SD) and cancer mortality [22]. The corresponding E-value would have to be 1.3 for cardiovascular mortality and 1.5 for other causes and total mortality as the outcome. The cohort was restricted to white Caucasian men, and the results from the present study may not be generalizable to female and other ethnicities. Previous interaction analysis by sex, however, has not revealed any gender differences [9].

## Conclusions

In a cohort of initially healthy men followed for over 30 years, we demonstrated that serum potassium, including the normal range, was positively and linearly associated with cancer death. Death due to cardiovascular and other causes were associated with potassium in the higher end of the scale. The strengths of the associations suggest possible clinical implications such as medical surveillance strategies for individuals with high levels of serum potassium.

## Supplementary Information

**Additional file 1: Table S1.** Other causes of death than cancer and cardiovascular disease, according to *European Shortlist for Causes of Death, 2012*, *n*=536. **Table S2.** Difference in restricted mean survival time (∆ RMST) in years of all-cause death for middle (4.1-4.5) and high (≥4.6) levels compared to low (≤4.0 mmol/L) levels of potassium at baseline. **Figure S1.** Hazard ratio (HR) for cancer death by levels of potassium stratified by smoking status at baseline. **Figure S2.** Hazard ratio (HR) for death related to other causes than cancer and cardiovascular disease by levels of potassium at baseline. **Figure S3.** Restricted mean survival time (RMST) for all-cause death for three categories (low [≤4.0], middle [4.1-4.5], and high [≥4.6] mmol/L)) of potassium at baseline.

## Data Availability

Data are from the *Oslo Ischemia Study*. Public availability would compromise privacy of the respondents. According to the approval from the Norwegian Regional committees for medical and health research ethics, the data is to be stored properly and in line with the Norwegian Law of privacy protection. Aggregated data are available on request. Please contact the corresponding author.

## Abbreviations

BMI: Body Mass Index
CI: Confidence Interval
HR: Hazard Ratio
ICD: International Classification of Diseases
RMST: Restricted Mean Survival Time
SD: Standard Deviation
