# Supplementary material for: Fasting serum potassium and long-term mortality in healthy men
Source: BMC Public Health. 2021 Apr 13;21:711. doi: 10.1186/s12889-021-10738-4 (PMC8045339; doi:10.1186/s12889-021-10738-4)
Supplement: Supplementary file 1 — Additional file 1: Table S1. Other causes of death than cancer and cardiovascular disease, according to European Shortlist for Causes of Death, 2012, n=536. Table S2. Difference in restricted mean survival time (∆ RMST) in years of all-cause death for middle (4.1-4.5) and high (≥4.6) levels compared to low (≤4.0 mmol/L) levels of potassium at baseline. Figure S1. Hazard ratio (HR) for cancer death by levels of potassium stratified by smoking status at baseline. Figure S2. Hazard ratio (HR) for death related to other causes than cancer and cardiovascular disease by levels of potassium at baseline. Figure S3. Restricted mean survival time (RMST) for all-cause death for three categories (low [≤4.0], middle [4.1-4.5], and high [≥4.6] mmol/L)) of potassium at baseline. [file 12889_2021_10738_MOESM1_ESM.docx]

## Supplementary Material

BMC Public Health

**Fasting serum potassium and long-term mortality in healthy men**

Falk RS, Robsahm TE, Paulsen JE, Stocks T, Drake I, Heir T.

Corresponding author:

Ragnhild S Falk, Oslo Centre for Biostatistics and Epidemiology, Oslo University Hospital r.s.falk@medisin.uio.no

**Table S1** Other causes of death than cancer and cardiovascular disease, according to *European Shortlist for Causes of Death, 2012*, n=536

**Table S2** Difference in restricted mean survival time (∆ RMST) in years of all-cause death for middle (4.1-4.5) and high (≥4.6) levels compared to low (≤4.0 mmol/L) levels of potassium at baseline

**Figure S1** Hazard ratio (HR) for **cancer death** by levels of potassium stratified by smoking status at baseline

**Figure S2** Hazard ratio(HR) for death related to **other causes** than cancer and cardiovascular disease by levels of potassium at baseline

**Figure S3** Restricted mean survival time (RMST) for all-cause death for three categories (low [≤4.0], middle [4.1-4.5], and high [≥4.6] mmol/L)) of potassium at baseline

**Table S1** Other causes of death than cancer and cardiovascular disease, according to *European Shortlist for Causes of Death, 2012*, n=536

| Cause of death | No. | (%) |
| --- | --- | --- |
| 1 Infectious and parasitic diseases | 18 | (3) |
| \| 3 Diseases of the blood and blood-forming organs and certain disorders involving the immune mechanism \|  \| \| --- \| --- \| | 4 | (.8) |
| 4 Endocrine, nutritional and metabolic diseases | 30 | (6) |
| 5 Mental and behavioral disorders | 37 | (7) |
| 6 Diseases of the nervous system and the sense organs | 60 | (11) |
| 8 Diseases of the respiratory system | 175 | (33) |
| 9 Diseases of the digestive system | 53 | (10) |
| 10 Diseases of the skin and subcutaneous tissue | 2 | (.4) |
| 11 Diseases of the musculoskeletal system/connective tissue | 9 | (2) |
| 12 Diseases of the genitourinary system | 24 | (4) |
| 15 Congenital malformations and chromosomal abnormalities | 2 | (.4) |
| 16 Symptoms, signs, ill-defined causes | 47 | (9) |
| 17 External causes of morbidity and mortality | 75 | (14) |

Reference:<https://ec.europa.eu/eurostat/ramon/nomenclatures/index.cfm?TargetUrl=LST_NOM_DTL&StrNom=COD_2012&StrLanguageCode=EN&IntPcKey=&StrLayoutCode=HIERARCHIC&IntCurrentPage=1>

**Table S2** Difference in restricted mean survival time (∆ RMST) in years of all-cause death for middle (4.1-4.5) and high (≥4.6) levels compared to low (≤4.0 mmol/L) levels of potassium at baseline

| Years of follow-up | Level of potassium (mmol/L) | Age  adjusted  ∆ RMST (95% CI) | | Multivariable adjusted*  ∆ RMST (95% CI) | |  |
| --- | --- | --- | --- | --- | --- | --- |
| 10 | Middle (4.1-4.5) | | -.1 (-.2 to .03) | | -.03 (-.1 to .1) | |
| 10 | High (≥4.6) | | -.1 (-.2 to .06) | | -.03 (-.2 to .1) | |
| 20 | Middle (4.1-4.5) | | -.3 (-.6 to .1) | | -.04 (-.4 to .4) | |
| 20 | High (≥4.6) | | **-.6 (-1.0 to -.1)** | | -.3 (-.8 to .2) | |
| 30 | Middle (4.1-4.5) | | **-.8 (-1.6 to -.1)** | | -.5 (-1.2 to .2) | |
| 30 | High (≥4.6) | | **-1.8 (-2.7 to -.9)** | | **-1.3 (-2.2 to -.4)** | |
| 40 | Middle (4.1-4.5) | | **-1.5 (-2.5 to -.5)** | | **-1.1 (-2.2 to -.1)** | |
| 40 | High (≥4.6) | | **-3.1 (-4.3 to -1.9)** | | **-2.5 (-3.7 to -1.2)** | |

∆ RMST = corresponds to the life year lost expressed in years compared to low levels of potassium

CI=confidence interval

*Adjusted for age, body mass index, smoking, systolic blood pressure, fitness, fasting blood glucose, sodium, triglycerides, cholesterol, creatinine and sedimentation rate

**Figure S1** Hazard ratio (HR) for cancer death by levels of potassium stratified by smoking status at baseline

**Figure S2** Hazard ratio(HR) for death related to other causes than cancer and cardiovascular disease by levels of potassium at baseline

**Figure S3** Restricted mean survival time (RMST) for all-cause death for three categories (low [≤4.0], middle [4.1-4.5], and high [≥4.6] mmol/L)) of potassium at baseline
